# Celecoxib Enhances the Chemotherapeutic Response of Cisplatin and TNF-α in SiHa Cells through Reactive Oxygen Species-Mediated Mitochondrial Pathway

**Published:** 2007-09

**Authors:** Meenakshi Kuhar, Sabiha Imran, Neeta Singh

**Affiliations:** 1 *Department of Biochemistry, All India Institute of Medical Sciences, New Delhi, India*

**Keywords:** cervical cancer, apoptosis celecoxib, cisplatin, TNF-α, mitochondria

## Abstract

Recently, many studies have shown that celecoxib induces apoptosis in various cancer cells by different mechanisms depending on the cell type. This study examined the effect of the selective COX-2 inhibitor celecoxib on cisplatin and TNF-α cytotoxicity and studied the role of mitochondria in the induction of apoptosis in the human cervical carcinoma SiHa cells. Apoptosis was detected by flow cytometry. The protein expression of Bcl-2, Bcl-X_L_, Bax, cytochrome c and AIF was analyzed by Western Blotting. The mRNA level of anti-oxidant enzymes was quantitated by RT-PCR. Priming SiHa cells with celecoxib increased the cisplatin-induced apoptosis by 20.56% and priming with celecoxib increased the TNF-α induced apoptosis by 22.07%. This was accompanied by downregulation of Bcl-X_L_ and Bcl-2 and upregulation of Bax. Cytosolic cytochrome c increased by 43.0% with celecoxib and TNF-α treatment but was not significant with celecoxib and cisplatin treatment. Nuclear AIF increased by 21.0% with celecoxib and cisplatin treatment whereas it was not significant with celecoxib and TNF-α treatment. The mRNA level of Mn-Superoxide dismutase, CuZn-Superoxide dismutase, Glutathione peroxidase and Catalase decreased significantly on priming with celecoxib and then treating with cisplatin or TNF-α. There was no significant increase in the activity of caspase-3 with either celecoxib or TNF-α treatment or with celecoxib and cisplatin treatment. The findings suggest that priming with celecoxib induces the TNF-α and cisplatin-mediated apoptosis in SiHa cells perhaps through ROS-mediated mitochondrial pathway.

## INTRODUCTION

Cervical cancer is one of the most common cancers amongst women. Despite its frequency and recurrence, the death rate has been declining over the past 40 years, due to early detection and treatment (1). Currently, the standard therapy for cervical carcinoma, following adequate radical surgery is simultaneous radiochemotherapy with a platinous chemotherapeutic agent. However, one disadvantage is that a number of patients have to break off therapy because of treatment-related toxicities (2). Cisplatin is among the most widely used and most effective chemotherapeutic agent for many types of human cancers. Because killing cancer cells by chemotherapy is principally executed by apoptosis, a detailed understanding of the apoptotic program and the factors that might influence cell death are essential for designing novel and more effective drug targets with relatively lesser side effects.

Many natural and synthetic compounds have been shown to modulate cisplatin and TNF-α mediated cytotoxicity in human cervical carcinoma cells. Vitamin C has been shown to enhance cisplatin-mediated apoptosis in human cervical carcinoma cells by downregulating transcription factor AP-1 and stabilizing P53 (3). Tumor necrosis factor-alpha activates both cell death and cell survival pathways, which render most cancer cells resistant to its cytotoxicity. Luteolin, a plant flavonoid, greatly sensitized TNF-α induced apoptotic cell death in a number of human cancer cell lines including cervical cancer cells by inhibiting TNF-α induced activation of nuclear transcription factor-kappa B, the main survival factor in TNF-α signaling (4).

The enzyme cyclooxygenase-2 (COX-2) has been associated with carcinogenesis and overexpression of COX-2 protein has been reported in a number of cancers including hepato-cellularcarcinoma (5), gastric cancer (6), esophageal cancer (7) colon cancer (8) and cervical carcinoma (9). COX-2 is also involved in apoptosis resistance, angiogenesis, and tumor cell invasiveness, which appear to contribute to its effects in tumorigenesis (10). Its relationship to apoptosis in cervical carcinoma patients treated with neoadjuvant chemotherapy has important clinical implications. An increased expression of COX-2 is associated with poor response to chemotherapy (11).

Celecoxib is a selective cyclooxygenase-2 (COX-2) inhibitor and non-steroidal anti-inflammatory drug (NSAID), which has been shown to be capable of inhibiting the growth of various cancer cell lines. This growth inhibitory effect was found to be independent of the COX-2 protein expression level of the cells lines (12). In the present study we investigated the enhancement of cytotoxic effects of cisplatin and TNF-α by selective COX-2 inhibitor celecoxib on human cervical cancer cells. We also investigated the role of mitochondrial pathway of apoptosis in the celecoxib-enhanced cytotoxic effects of cisplatin and TNF-α.

## MATERIALS AND METHODS

### Cell Culture and Treatments

Human Cervical Carcinoma cells SiHa were grown in DMEM medium supplemented with 10% fetal calf serum and containing antibiotics (100 U/ml penicillin, 0.1 mg/ml streptomycin) in a humidified atmosphere of 5% CO_2_ in air at 37°C. Logarithmically growing cells were used for all experiments. Cells were treated with standardized doses of celecoxib (100 μM) for 24 hours followed by cisplatin (5 μg/ml) for another 24 hours. Cells were also treated with celecoxib (100 μM) for 24 hours followed by TNF-α (5 ng/ml) for another 24 hours.

### Isolation of Nuclei, Mitochondria and Cytosol

Cells were sonicated in buffer containing 10 mM Tris-HCl pH7.5, 10 mM NaCl and 1.5 mM MgCl_2_, 175 mM Sucrose and 12.5 mM EDTA and the cell extract centrifuged at 1000 g for 10 minutes to pellet the nuclei. The supernatant thus obtained was centrifuged at 18,000 g for 30 minutes to pellet the mitochondria. The mitochondria were purified as described (13). The resulting supernatant was termed as the cytosolic fraction. The pellets were lysed and protein was estimated in all the three fractions by Bradford’s method (14). The purity of the fractions was confirmed by assaying the marker enzymes succinate dehydrogenase for mitochondria, lactate dehydrogenase for the cytosol and NMN adenylyl transferase for the nuclear fraction.

### Western Blotting

The whole cells, nuclei or the mitochondrial fractions were lysed in ice-cold lysis buffer (50 mM Tris, 150 mM NaCl, 2.5 mM EDTA, 0.1% SDS, 0.5% sodium deoxycholate, 1% NP-40 and 0.02% sodium azide) containing protease inhibitors as described (15). After incubation for 30 minutes on ice, samples were centrifuged at 10,000 rpm for 30 minutes. Protein content in the lysed extracts was determined using the Bradford’s method. Equal amounts of protein (80 μg/lane) was loaded on 10-12% SDS-polyacrylamide gels and transferred to nitrocellulose membranes. The primary antibodies against Bcl-X_L_, Bcl-2, Bax, cytochrome c and AIF were obtained from Santa Cruz Biotechnology USA. The secondary antibody used was the appropriate alkaline phosphatase conjugated antimouse, antirabbit or antigoat IgG (Promega, Madison, WI). Proteins were detected using BCIP-NBT substrate from Promega. Densitometric scanning was performed using Alpha Imager 2200. Positive and negative controls were run with each antibody.

### Flow Cytometry

Apoptosis was measured by flow cytometry as described earlier (16). In brief the cells were washed twice with PBS and fixed in 70% ethanol overnight. Cells were then washed twice with PBS to remove ethanol and then incubated in propidium iodide (20 μg/ml) for 1 hour. Flow cytometry was performed using an EPICS XL-MCL flow cytometer (Coulter Electronics,Miami,FL) software. Win MDI 2.8 Software was used to generate histograms, which were then used to determine the cell cycle phase distribution after debris exclusion. The sub G_1_-G_0_ cell fraction was considered as representative of apoptotic cells.

### Reverse Transcriptase mediated-PCR

The mRNA levels of various enzymes related to oxidative stress were analyzed by RT-PCR. RNA was isolated using TRI-Reagent from Sigma and precipitated by isopropanol as described earlier (17). The precipitated RNA was dissolved in DEPC-water. The purity of the isolated RNA was checked by running it on formaldehyde gel and quantitated by taking absorbance at 260 nm and 280 nm. The RT–Reaction mixture was incubated at 42°C for 10 min to disrupt the secondary structure in the template of RNA.This was followed by the addition of reverse transcriptase enzyme from Stratagene and incubation at 37°C for 60 min for the synthesis of cDNA, which was then used as a template for PCR using primers specific for Mn-Superoxide Dismutase, Cu-Zn Superoxide Dismutase, Glutathione Peroxidase and Catalase mRNA.

### Manganese superoxide dismutase

- Forward primer: 5’GGCCTGATTATCTAAAAGCTATTTGG 3’
- Reverse primer: 5’CGATCGTGGTTTACTTTTTGCA 3’

### Copper-Zinc superoxide dismutase

- Forward primer: 5’TGGTGGTCCATGAAAAAGCA 3’
- Reverse primer: 5’CCAGCGTTTCCTGTCTTTGTACT 3’

### Glutathione peroxidase

- Forward primer: 5’TGTGCCCCTACGGAGGTAC 3’
- Reverse primer: 5’AGCTGGGCCCTTGAGACAG 3’

### Catalase

- Forward primer: 5’AGAGGAAACGTCTGTGTGAGAACA 3’
- Reverse primer: 5’TGACCGCTTTCTTCTGGATGA 3’

### Bcl-2

- Forward primer: 5’GCCGGTTCAGGTACTCAGTCA 3’
- Reverse primer: 5’CATGTGTGTGGAGAGCGTCAA 3’

### Caspase-3 assay

Caspase-3 activity was measured by the direct assay of enzyme activity in cell lysates using synthetic fluorogenic substrate Ac-DEVD-AMC. Cells were washed with PBS, pH7.5 and lysed in 10 mM Tris HCl, pH7.5, 130 mM NaCl, 1% Triton X-100, 10 mM Na_4_P_2_O_7_ and 10 mM, Na_2_HPO_4_ on ice (10 × 10^6^ cells/ml of lysis buffer). 100 μl of cell lysate was added to the reaction buffer (20 mM HEPES, pH7.2; 10% Glycerol; 2 mM DTT; 250 μM Ac-DEVD-AMC) and incubated at 37°C for one hour. Amounts of fluorogenic moiety released were measured using a spectrofluorimeter with excitation at 380nm and emission at 420-460 nm (18).

### Statistical Analysis

Each experiment was repeated three times. All data are expressed as mean ± *SD*. Students *t* test was used to determine the significance between the control and various experimental groups. A difference was considered statistically significant at *p*<0.05.

## RESULTS

### Celecoxib enhanced cisplatin and TNF- α induced cell death in SiHa cells

**Figure 1:**
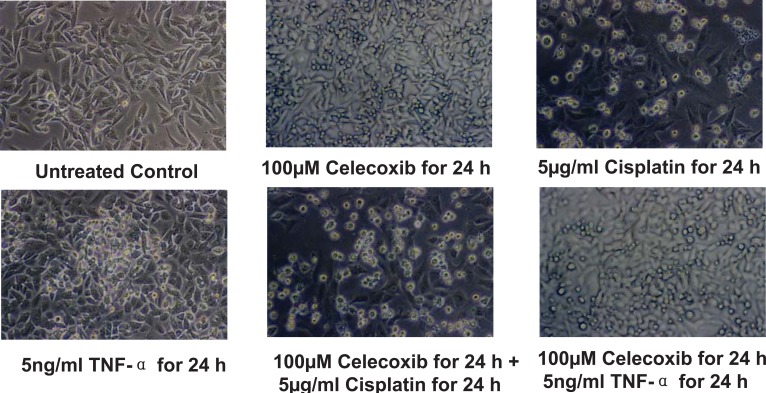
Effect of priming with celecoxib on cisplatin and TNF-α induced apoptosis in NCI-SiHa cells as seen by morphology.

**Figure 2:**
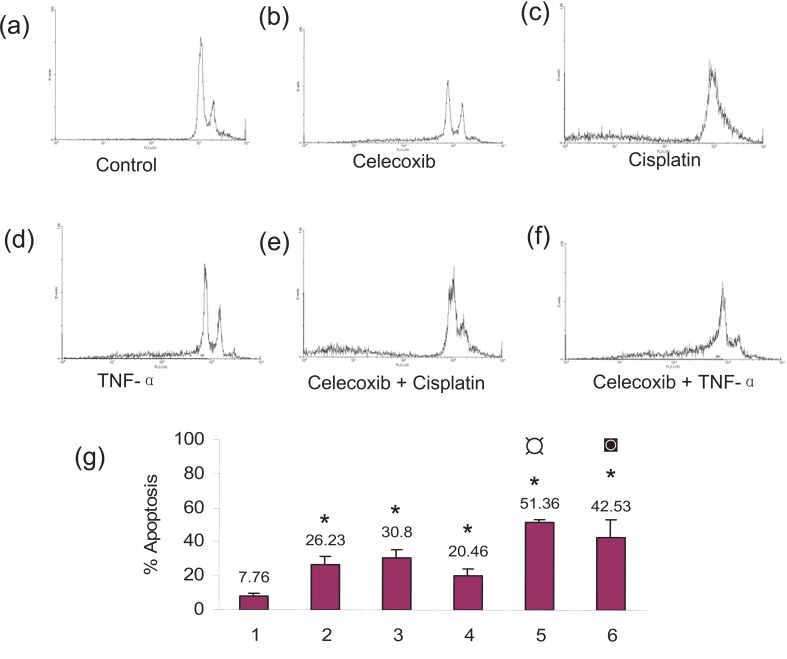
Effect of priming with celecoxib on cisplatin and TNF-α induced apoptosis in SiHa cells as quantitated by Flow Cytometry. (a) Untreated control cells; (b) 100 μM celecoxib for 24 h; (c) 5μg/ml cisplatin for 24 h; (d) 5ng/ml TNF-α for 24 h; (e) 100μM celecoxib for 24 hours followed by 5μg/ml cisplatin for another 24 hours; (f) 100 μM celecoxib for 24 hours followed by 5ng/ml TNF-α for another 24 hours; (g) (**1**) Untreated Control cells (**2**) 100 μM celecoxib for 24 h (**3**) 5 μg/ml cisplatin for 24 h (**4**) 5 ng/ml TNF-α for 24 h (**5**) 100 μM celecoxib for 24 hours followed by 5μg/ml cisplatin for another 24 hours (**6**) 100 μM celecoxib for 24 hours followed by 5ng/ml TNF-α for another 24 hours. The data represents the average value obtained from three independent experiments. The *bars* represent mean ± S.D. *p*<0.05. *, indicates the significance between treated samples and the untreated control; ¤, indicates the significance between (celecoxib+cisplatin) treatment and cisplatin alone; ◙, indicates the significance between (celecoxib+ TNF-α) treatment and TNF-α alone. Figure 2(a)-(f) is the representation of a single independent experiment and figure 2(g) shows mean of the results obtained from three independent experiments.

Apoptosis was observed by morphological changes such as cell shrinkage, rounding of the cells and formation of apoptotic bodies. This was more prominent for the combination treatments (Figure 1). The untreated control cells showed 7.76% apoptosis. Celecoxib by itself caused 26.23% apoptosis, cisplatin by itself induced 30.80% apoptosis and TNF-α by itself induced 20.46% apoptosis. Pretreatment with celecoxib increased cisplatin-induced apoptosis to 51.36% and that of TNF-α to 42.53% (Figure 2). The morphological changes were in agreement with the flow cytometry data.

### Bcl-2 was downregulated by celecoxib pretreatment

**Figure 3:**
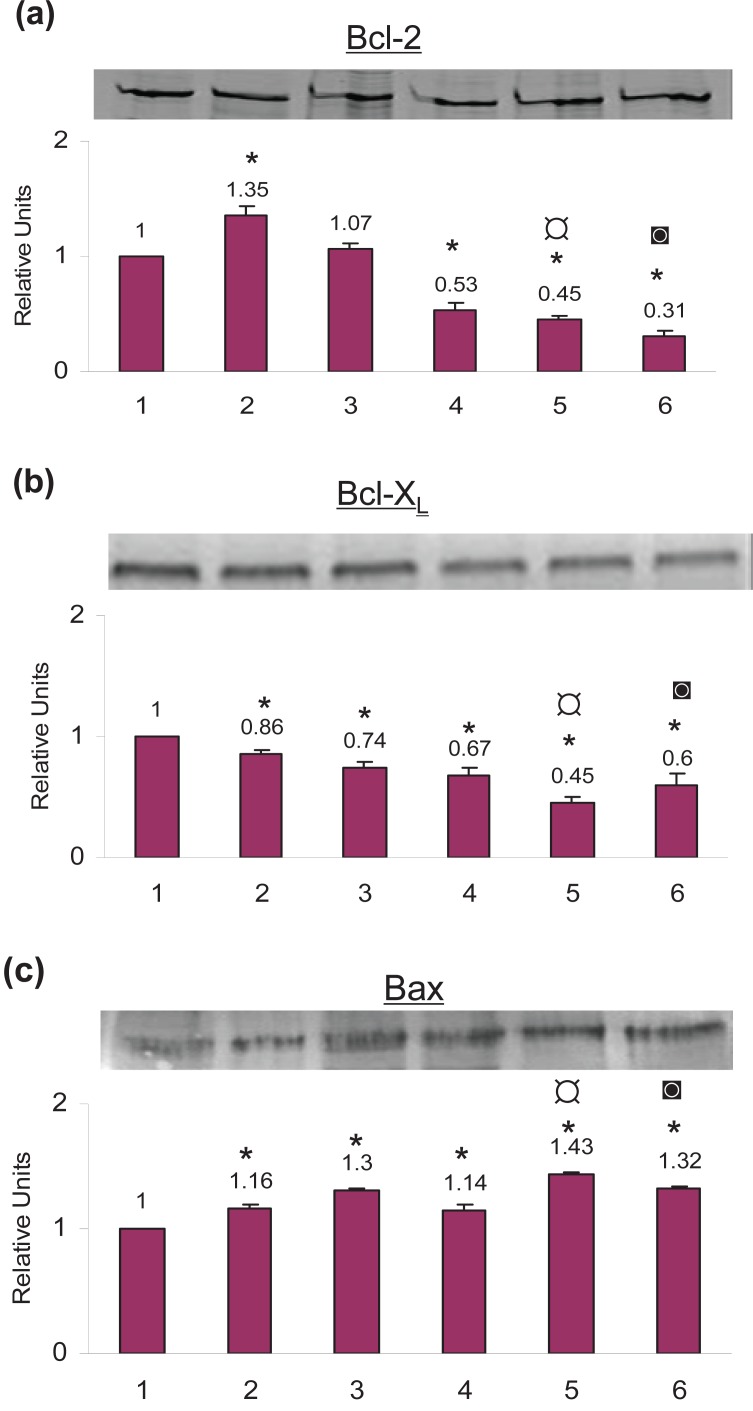
Protein expressions of (a) Bcl-2 (b) Bcl-X_L_ and (c) Bax in SiHa cells as analyzed by Western Blotting. The cells were treated with celecoxib followed by cisplatin and TNF-α as described and subjected to Western Blotting. (**1**) Untreated Control cells (**2**) 100 μM celecoxib for 24 h (**3**) 5μg/ml cisplatin for 24 h (**4**) 5 ng/ml TNF-α for 24 h (**5**) 100 μM celecoxib for 24 hours followed by 5 μg/ml cisplatin for another 24 hours (**6**) 100 μM celecoxib for 24 hours followed by 5 ng/ml TNF-α for another 24 hours. The data represents the average value obtained from three independent experiments. The bands shown are the representation of a single independent experiment. The *bars* represent mean ± S.D. *p*<0.05. *, indicates the significance between treated samples and the untreated control; ¤, indicates the significance between (celecoxib+cisplatin) treatment and cisplatin alone; ◙, indicates the significance between (celecoxib+ TNF-α) treatment and TNF-α alone.

Celecoxib treatment by itself increased Bcl-2 protein level by 35.0% as compared to the control cells. Cisplatin treatment increased Bcl-2 by 7.0% (not significant) and TNF-α by 47.0% as compared to the control cells. On priming with celecoxib followed by treatment with cisplatin the Bcl-2 decreased by 55.0% and by 69.0% on TNF-α treatment as compared to the control cells (Fig. 3a).

### Bcl-X_L_ was downregulated by celecoxib pretreatment

By itself celecoxib treatment decreased Bcl-X_L_ by 14.0%, cisplatin by 26.0% and TNF-α by 33.0% as compared to the control cells. When cells were primed with celecoxib and then treated with cisplatin the Bcl-X_L_ level decreased by 55.0% whereas when treated with TNF-α the Bcl-X_L_ decreased by 40.0% (Fig. 3b).

### Bax was upregulated by celecoxib pretreatment

Celecoxib treatment by itself increased Bax by 16.0%, cisplatin treatment increased Bax by 30.0% and TNF-α treatment by 14.0% as compared to the control cells. When cells were primed with celecoxib and then treated with cisplatin the Bax increased by 43.0% and with TNF-α it increased by 32.0% as compared to the control cells (Fig. 3c).

### No Translocation of cytochrome c to the cytosol by celecoxib pretreatment

**Figure 4:**
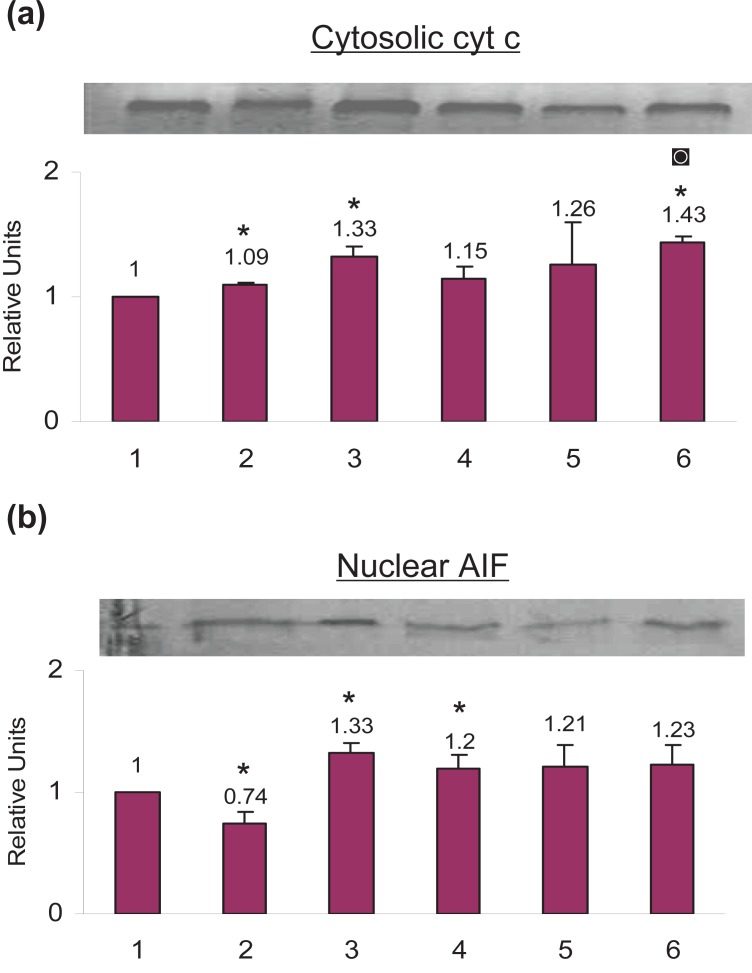
Effect of celecoxib priming on the translocation of (a) cytochrome c and (b) Apoptosis Inducing Factor (AIF) in SiHa cells as analyzed by Western Blotting. (**1**) Untreated Control cells (**2**) 100 μM celecoxib for 24 h (**3**) 5μg/ml cisplatin for 24 h (**4**) 5ng/ml TNF-α for 24 h (**5**) 100 μM celecoxib for 24 hours followed by 5μg/ml cisplatin for another 24 hours (**6**) 100 μM celecoxib for 24 hours followed by 5 ng/ml TNF-α for another 24 hours. The data represents the average value obtained from three independent experiments. The bands shown are the representation of a single independent experiment. The *bars* represent mean ± S.D. *p*<0.05. *, indicates the significance between treated samples and the untreated control; ◙, indicates the significance between (celecoxib+ TNF-α) treatment and TNF-α alone.

Treatment with celecoxib increased cytosolic cytochrome c by 9.0%, cisplatin by 33.0% and TNF-α by 15.0% as compared to the control cells. When cells were primed with celecoxib and then treated with cisplatin the cytosolic cytochrome c increased by 26.0% whereas when cells were primed with celecoxib and then treated with TNF-α the cytosolic cytochrome c increased by 43.0% as compared to the control cells (Fig. 4a).

### AIF is not involved in the celecoxib- or TNF-mediated apoptosis

Celecoxib treatment decreased nuclear AIF by 26.0%, cisplatin treatment increased nuclear AIF by 33.0% and TNF-α treatment increased nuclear AIF by 20.0% as compared to the control cells, which was not significant. When cells were primed with celecoxib and then treated with cisplatin the nuclear AIF increased by 21.0% whereas when cells were primed with celecoxib and treated with TNF-α the nuclear AIF increased by 23.0% as compared to the control cells which was not significant (Fig. 4b).

### Anti-oxidant enzymes at the mRNA level

**Figure 5:**
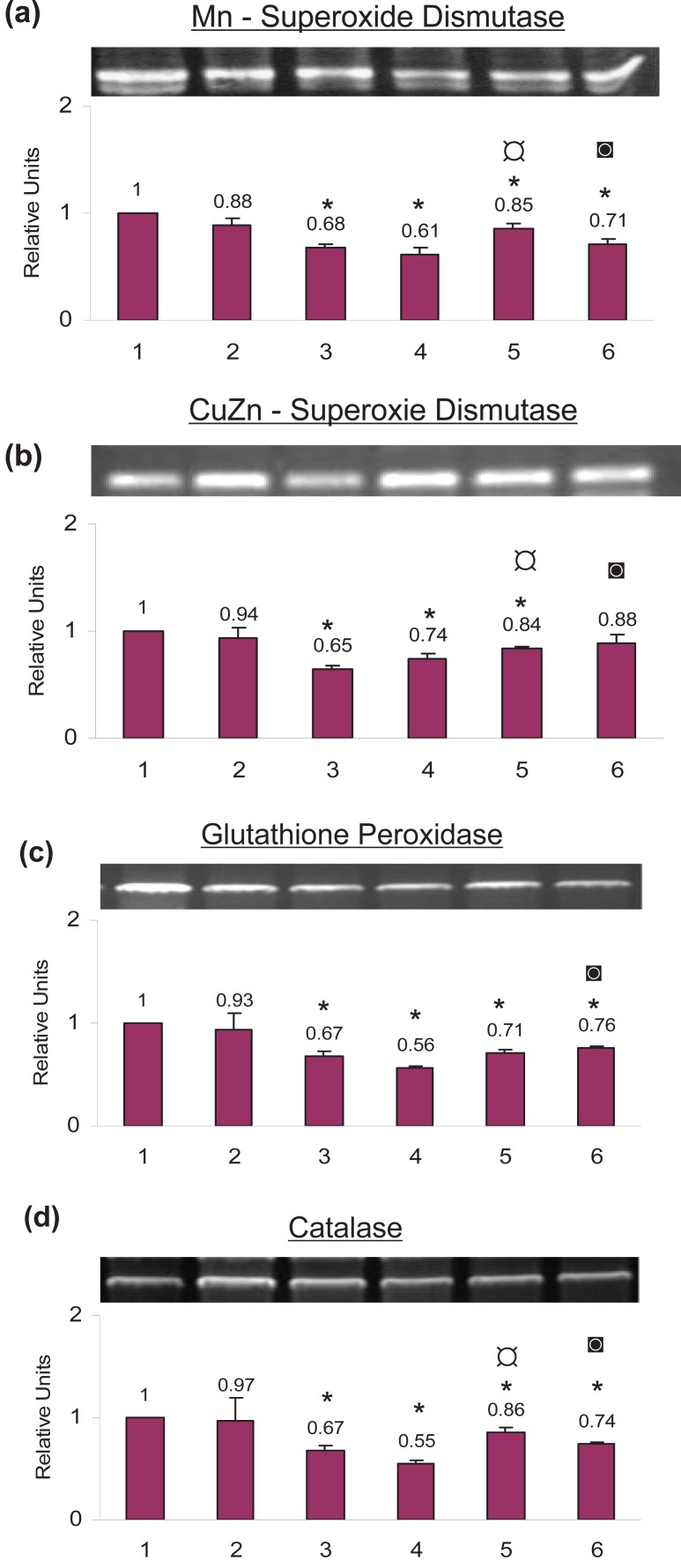
Effect of celecoxib priming on the mRNA expression of anti-oxidative enzymes in SiHa cells as analyzed by RT-PCR. The cells were treated with celecoxib followed by cisplatin and TNF-α as described. RNA was isolated and the various anti-oxidative enzymes were quantitated by RT-PCR. (**1**) Untreated Control cells (**2**) 100 μM celecoxib for 24 h (**3**) 5 μg/ml cisplatin for 24 h (**4**) 5 ng/ml TNF-α for 24 h (**5**) 100 μM celecoxib for 24 hours followed by 5μg/ml cisplatin for another 24 hours (**6**) 100 μM celecoxib for 24 hours followed by 5 ng/ml TNF-α for another 24 hours. The data represents the average value obtained from three independent experiments. The bands shown are the representation of a single independent experiment. The *bars* represent mean ± S.D. *p*<0.05. *, indicates the significance between treated samples and the untreated control; ¤, indicates the significance between (celecoxib+cisplatin) treatment and cisplatin alone; ◙, indicates the significance between (celecoxib+ TNF-α) treatment and TNF-α alone.

**Mn-Superoxide dismutase.** The mRNA level of Mn-Superoxide dismutase decreased significantly with cisplatin (34.0%), TNF-α (39.0%), celecoxib+cisplatin (15.0%) and celecoxib + TNF-α (29.0%) treatment. The decrease with celecoxib alone (12.0%) was not significant as compared to the control cells (Fig. 5a).

**CuZn-Superoxide dismutase.** The mRNA level of CuZn-Superoxide dismutase decreased by 35.0% with cisplatin treatment alone, 26.0% with TNF-α treatment alone and 16.0% with celecoxib+cisplatin treatment. The change with celecoxib alone or with celecoxib+ TNF- α treatment was not significant as compared to the control cells (Fig. 5b).

**Glutathione peroxidase.** Glutathione peroxidase mRNA decreased significantly with cisplatin (33.0%), TNF-α (44.0%), celecoxib+cisplatin (29.0%) and celecoxib+ TNF- α (24.0%) as compared to the control cells. The increase with celecoxib alone (7.0%) was not significant as compared to the control cells (Fig. 5c).

**Catalase.** Catalase mRNA decreased significantly with cisplatin (33.0%), TNF-α (45.0%), celecoxib+cisplatin (14.0%) and celecoxib+ TNF-α (26.0%) as compared to the control cells. The decrease with celecoxib alone (3.0%) was not significant as compared to the control cells (Fig. 5d).

**Figure 6:**
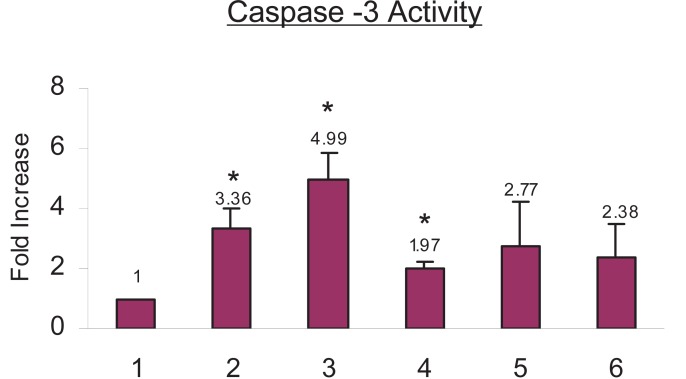
Caspase-3 activity as a result of celecoxib priming in SiHa cells. The cells were treated with celecoxib followed by cisplatin and TNF-α as described. The activity of the enzyme caspase-3 was measured using Acetyl-DEVD-AMC as the substrate. (**1**) Untreated Control cells (**2**) 100 μM celecoxib for 24 h (**3**) 5 μg/ml cisplatin for 24 h (**4**) 5 ng/ml TNF-α for 24 h (**5**) 100 μM celecoxib for 24 hours followed by 5 μg/ml cisplatin for another 24 hours (**6**) 100 μM celecoxib for 24 hours followed by 5 ng/ml TNF-α for another 24 hours. The data represents the average value obtained from three independent experiments. The *bars* represent mean ± S.D. *p*<0.05. *, indicates the significance between treated samples and the untreated control.

**Celecoxib priming does not result in caspase-3 activation.** Celecoxib treatment increased caspase-3 activity by 3.36 fold, cisplatin treatment increased caspase-3 activity by 4.99 fold and TNF-α treatment increased caspase-3 activity by 1.97 fold as compared to the control cells. When cells were primed with celecoxib and then treated with cisplatin, caspase-3 activity increased by 2.77 fold as compared to the control cells which was not significant. Whereas when cells were primed with celecoxib and treated with TNF-α caspase-3 activity increased by 2.38 fold, as compared to the control cells which were not significant (Fig. 6).

## DISCUSSION

Cervical carcinoma is a human papillomavirus (HPV)-associated cancer for which treatment options still mainly rely on surgical procedures, with or without adjuvant radiotherapy and chemotherapy (19). Recent advances in cancer chemotherapy have drawn the attention of investigators to the usefulness of chemotherapy for cancer of the uterine cervix. Though satisfactory therapeutic results have been achieved with cisplatin-based cyclic balloon-occluded arterial infusion chemotherapy (BOAI), nevertheless, there have been some patients in whom it was ineffective and these patients showed significantly higher expression of COX-2 after BOAI, accompanied by cancer cell apoptosis inhibition. It has been suggested that overexpression of COX-2 inhibits cancer cell apoptosis and adversely influences the prognosis (20). Thus assessment of the COX-2 status could be useful to identify cervical cancer patients who may benefit from neoadjuvant chemotherapy (21). In cervical cancer, celecoxib treatment decreases tumor COX-2 expression and markers of proliferation and neoangiogenesis, thus suggesting that selective COX-2 inhibitors may be a promising strategy not only for chemopreventive approaches but also for therapeutic approaches in cervical cancer (22).

Tumor necrosis factor- alpha (TNF-α) induces apoptosis of a variety of tumor cell types. Celecoxib induces apoptosis independent of COX-2 activity. Caspase-8 and -9 are implicated in the apoptotic effect of celecoxib in cervical cancer cells. We investigated the effect of the selective COX-2 inhibitor celecoxib on cisplatin and TNF-α cytotoxicity and studied the role of the mitochondrial pathway in the induction of apoptosis in SiHa cells. In the present study a 100 μM dose of celecoxib induced significant apoptosis in SiHa cells and it enhanced both the cisplatin and TNF-α mediated apoptosis. This was accompanied by a decrease in the expression of Bcl-2 and Bcl-X_L_ and an increase in the Bax protein levels. Bcl-2 and Bcl-X_L_ exert their anti-apoptotic effect, at least in part by binding to Bax and related pro-apoptotic proteins. A higher expression of the Bax protein and gene has been associated with accelerated cancer cell apoptosis. On the other hand, the patients in whom cisplatin-based therapy was ineffective showed higher expression of the Bcl-X_L_ protein and gene (23).

Because Bcl-2 family proteins can regulate release of cytochrome *c* and AIF from the mitochondria, the proteins Bcl-2 and Bcl-X_L_ play a significant role in the process of induction of apoptosis. Cytochrome c released from the mitochondria forms a complex with procaspase-9 and apoptotic protease-activating factor-1 (Apaf-1), resulting in activation of procaspase-9. In contrast, AIF is believed to be responsible for chromatin condensation and is usually associated with caspase-independent cell death. Similar to cytochrome *c*, AIF is released from the mitochondria in response to death stimuli. On induction of apoptosis, AIF is translocated to the nucleus and causes large-scale DNA fragmentation and chromatin condensation in a caspase-independent manner (24). There was no significant change in the cytosolic cytochrome c and nuclear AIF levels with the combination treatment of celecoxib and cisplatin. Further the caspase-3 activity was also not significantly increased.

There was a significant increase in the cytosolic cytochrome c (43.0%) with the combination treatment of celecoxib and TNF. This observed increased cytochrome c release accompanied but non significant increase in caspase-3 activity implies that cytochrome c does not mediate further downstream events in the apoptotic pathway. Probably there is an inhibition at the level of caspase activation by Inhibitor of Apoptosis Proteins (IAPs). Nuclear AIF was not significantly altered with the combination treatment of celecoxib and TNF-α implicating the non-involvement of AIF in the apoptotic process.

Mitochondria are considered to be a major source of intracellular oxygen radicals. Accumulation of ROS results in cellular oxidative stress, and if not corrected, can lead to the damage of important biomolecules such as membrane lipids, proteins and DNA and its prolonged accumulation may cause irreversible cellular injury ultimately resulting in cell death. The mRNA levels of various enzymes related to oxidative stress showed significant decrease suggesting the role of oxygen radicals in the induction of apoptosis.

Given the substantial role of mitochondria in ATP metabolism, in generation of free radicals, and in the regulation of apoptosis, mitochondrial dysfunction seems likely to affect cellular energy capacities, increase oxidative stress, cause ROS-mediated damage to DNA and alter the cellular response to apoptosis induction by anticancer agents.

Arsenic trioxide is known to induce apoptosis in human cervical cancer cells through a reactive oxygen species-dependent pathway involving loss of the mitochondria membrane permeability (Δψ_m_) and caspase-3 activation (25). Precise mechanism of the ROS-mediated caspase-independent apoptotic cell death triggered by As_2_O_3_ treatment still remains unclear.

These results are consistent with previous reports that Bcl-2 can block loss of mitochondria membrane permeability and AIF release (26). In some systems, Bcl-2 (or Bcl-X_L_) appears to influence ROS generation (27) whereas in others it does not affect ROS production but does prevent oxidative damage to cellular constituents (28). It has also been proposed that Bcl-2 modulates level of ROS through induction of intracellular antioxidants. This activity of Bcl-2 may control entry into apoptosis (29).

Caspase activation is thought to be an important mechanism for the apoptotic cell death program; however, our findings suggest that cell death induced by the combination treatment of celecoxib and TNF-α in human cervical cancer cells is caspase-independent as there was no significant increase in of caspase-3 activity though the cytosolic cytochrome c was elevated. There appears to be a block at the level of caspase inhibition possibly by IAPs. Neither there was any significant change in the cytosolic cytochrome c and the caspase-3 activity with celecoxib and cisplatin treatment. Possibly a reactive oxygen-mediated caspase-independent pathway is involved. Such a mechanism of cell death has been recently reported in human cervical cancer cells (30).
